# Host-Microbe Multi-omic Profiling Identifies a Unique Program of COVID-19 Inflammatory Dysregulation in Solid Organ Transplant Recipients

**DOI:** 10.21203/rs.3.rs-3621844/v1

**Published:** 2023-12-20

**Authors:** Charles Langelier, Harry Pickering, Joanna Schaenman, Hoang Phan, Cole Maguire, Alexandra Tsitsiklis, Nadine Rouphael, Nelson Higuita, Mark Atkinson, Scott Breckenridge, Monica Fung, William Messer, IMPACC Network, Ramin Salehi-rad, Matthew Altman, Patrice Becker, Steven Bosinger, Walter Eckalbar, Annmarie Hoch, Naresh Jayavelu, Seunghee Kim-Schulze, Meagan Jenkins, Steven Kleinstein, Florian Krammer, Holden Maecker, Al Ozonoff, Joann Diray-Arce, Albert Shaw, Lindsey Baden, Ofer Levy, Elaine Reed

**Affiliations:** University of California, San Francisco; UCLA; UCLA; University of California, San Francisco; UT Austin; University of California, San Francisco; Emory University; U Oklahoma; University of Florida; U Washington; UCSF; OHSU; UCSF; UCLA; Benaroya Research Institute; National Institute of Allergy and Infectious Diseases/National Institutes of Health; Emory University; University of California, San Francisco; Harvard; Benaroya Research Institute; Icahn School of Medicine at Mount Sinai; UCLA; Yale University; Icahn School of Medicine at Mount Sinai; Stanford University School of Medicine; Boston Children’s Hospital; Harvard; Yale University School of Medicine; Harvard; Harvard Medical School; University of California Los Angeles

## Abstract

Coronavirus disease 2019 (COVID-19) poses significant risks for solid organ transplant (SOT) recipients, who have atypical but poorly characterized immune responses to SARS-CoV-2 infection. We sought to understand and the host immunologic and microbial features of COVID-19 in SOT recipients by leveraging a prospective multicenter cohort of 1164 hospitalized patients. Using multi-omic immuoprofiling, we studied 86 SOT recipients in this cohort, who were age- and sex-matched 2:1 with 172 non-SOT controls. PBMC and nasal transcriptional profiling unexpectedly demonstrated upregulation of innate immune pathways related to interferon (IFN) and Toll-like receptor signaling, and complement activation, in SOT recipients. Longitudinal analyses across the first 30-days post-hospitalization demonstrated persistent upregulation of these innate immunity pathways in SOT recipients. The levels of several proinflammatory serum chemokines, such as CX3CL1 and KITLG, were also higher in SOT recipients at the time of hospitalization, although IFN-gamma levels were lower. We observed differential dynamics of CXCL11, which remained persistently elevated in SOT recipients over the course of hospitalization. Nasal microbiome alpha diversity was higher in SOT recipients versus controls, but no differences in taxonomic abundance beyond SARS-CoV-2 were observed. SOT recipients had higher nasal SARS-CoV-2 viral loads and impaired viral clearance compared to controls. Antibody analysis demonstrated lower anti-SARS-CoV-2 spike IgG levels in SOT recipients upon hospitalization, but no distinctions over time compared to controls. Mass cytometry demonstrated marked differences in blood immune cell populations, with SOT recipients exhibiting decreased plasmablasts and transitional B cells, and increased senescent T cells. Severe disease in SOT recipients was characterized by a less robust induction of inflammatory chemokines, such as IL-6 and CCL7, and a more subtle proinflammatory transcriptional response in the blood and airway. Together, our study reveals distinct immune features and altered viral dynamics in SOT recipients compared to non-SOT controls. We unexpectedly find that SOT recipients exhibit an augmented, predominantly innate immune response in both the blood and upper respiratory tract that remains relatively stable across disease severity, in contrast to non-SOT controls. These findings may relate to the paradoxical observation that SOT recipients have similar COVID-19 mortality rates versus the general population, despite being more susceptible to SARS-CoV-2 infection, remaining infectious longer, and having higher rates of hospitalization. In summary, we find that COVID-19 in SOT recipients is characterized by a biologically distinct immune state, suggesting the potential for unique prognostic biomarkers and therapeutic approaches in this vulnerable population.

## Introduction

Coronavirus disease 2019 (COVID-19) has resulted in an enormous societal burden, with a toll of millions of infections and deaths worldwide^1^. Immunocompromised patients who have undergone solid organ transplantation (SOT) are more susceptible to SARS-CoV-2 infection and produce less robust antibody responses following vaccination^2^, although they can achieve effective T cell responses with multiple vaccinations^3^. In addition, they are more likely to be hospitalized, experience adverse clinical outcomes, and have longer durations of infectiousness compared to the general population^4–8^. Surprisingly, however, COVID-19 mortality in SOT recipients is not clearly higher compared to immunocompetent individuals, in propensity-matched studies^5,8–10^.

To prevent organ rejection, SOT depends on immune suppression with a battery of agents including calcineurin inhibitors (e.g., cyclosporin, tacrolimus), cell cycle inhibitors (e.g., mycophenolate mofetil), and corticosteroids. This leads to an altered immunological landscape in SOT recipients, resulting in differing host responses to severe infections, including from SARS-CoV-2. SOT recipients at baseline have decreased frequencies of circulating naïve T and NK cells, and an increased frequency of terminally differentiated and senescent T cells compared with the general population^11–13^. Few studies, however, have profiled the immune landscape of SOT recipients in the context of severe infection, and none have yet used a multi-omic approach to assess their responses at the cellular, protein, transcriptional, and microbial levels.

The distinct immune responses of SOT recipients could theoretically be both detrimental and advantageous in the context of COVID-19. On the one hand, impaired innate and adaptive immunity in SOT recipients increases susceptibility to infection and impairs viral clearance^8^, which could lead to worse outcomes. On the other hand, because severe COVID-19 is characterized by a dysregulated, overexuberant inflammatory response^14–16^, intrinsic immunosuppression of SOT recipients could confer protection against severe disease. Developing a better mechanistic understanding of this tenuous immune balance in SOT recipients could inform more effective treatment approaches for COVID-19 or other respiratory viral infections, in particular the optimal use of immune modulating therapies^8^.

Here, we leverage a multicenter cohort^16–18^ of 1164 vaccine-naïve patients hospitalized for COVID-19 to carry out the first longitudinal immunoprofiling analysis of host and microbe in SOT recipients with acute SARS-CoV-2 infection. This cohort afforded the unique opportunity to study immune responses over the course of hospitalization through concurrent analysis of transcriptional, proteomic, cellular, and antibody responses in addition to viral abundance and the airway microbiome. Contrary to expectations, we find that SOT recipients demonstrate a globally heightened innate inflammatory response compared to non-SOT controls, and observe that established biomarkers of COVID-19 severity do not correlate with disease trajectory in this vulnerable demographic.

## Results

### Patient cohort

**Figure 1:**
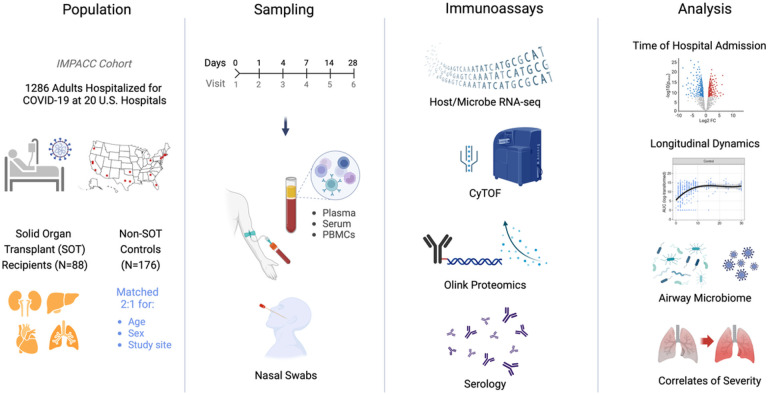
Study overview. This study evaluated solid organ transplant recipients (N = 86) matched 1:2 with non-transplant controls (N = 172) enrolled in the IMPACC cohort of patients hospitalized for COVID-19 at 20 medical sites across the United States. Blood (PBMCs and serum) and nasal swab samples were collected at up to 6 visits over 28 days, and processed for RNA sequencing, proximity extension assay (Olink) soluble proteomics, mass cytometry, and serology.

We conducted a case-control study of patients hospitalized for COVID-19 within the IMPACC cohort, comprised 1164 patients enrolled across the US^16–18^ between May 2020 and March 2021. 86 SOT recipients from 11 medical centers were matched 2:1 by age, sex, and study site with 172 non-SOT controls from the same cohort (Fig. 1, Table 1). The most common transplanted organ type was kidney (Supp. Table 1), with approximately equal representation of heart, liver, and lung. We found no differences between the two groups in terms of ICU admission, intubation status, or COVID-19 severity as measured by five established COVID-19 outcome trajectory groups (TG)^17^, or as measured by 28-day mortality. Trajectory groups (TG) 1–3 have mild to moderate disease based on hospital stay and level of respiratory support, while TG4 is characterized by longer hospitalizations and prolonged respiratory support requirements and TG5 by death within 28 days^17^.

To investigate host immunologic and microbial features associated with COVID-19 in SOT recipients, we assessed data from mass cytometry (CyTOF), transcriptional profiling, proteomics, and serologic analyses in the blood, as well as nasal swab transcriptional profiling and metatranscriptomics at hospital admission (i.e., Visit 1, which is within approximately 72 hours of hospital admission), and longitudinally at up to six timepoints up to approximately 28 days post-hospital admission (Fig. 1).

### SOT is associated with increased SARS-CoV-2 viral abundance, and impaired viral clearance

**Figure 2:**
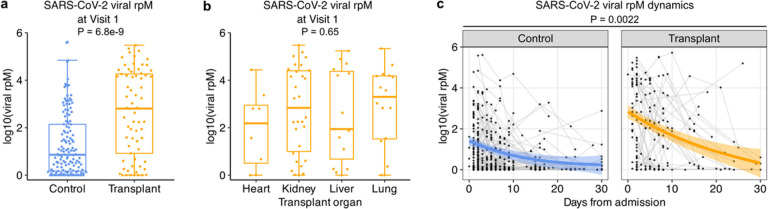
SOT recipients have higher SARS-CoV-2 viral rpM and impaired viral clearance compared to controls. (a, b) Box plots showing viral rpM at Visit 1 of (a) transplant and control groups, and (b) different organ transplant types. P-values were calculated with (a) a linear model or (b) likelihood ratio test. (c) Plot showing the dynamics of viral rpM up to 30 days after hospital admission of the transplant and control groups. P-value was calculated with a linear mixed effects model.

We began our analyses by examining the SARS-CoV-2 viral abundance, as measured in reads per million (rpM) by nasal metatranscriptomic RNA sequencing and N-gene reverse transcription PCR (Supp. Fig. 1). SOT recipients had significantly higher SARS-CoV-2 viral rpM at Visit 1 (Fig. 2a, P = 5.87e-9), which could not be explained by differences in time from symptom onset (P = 0.16, Supp. Fig. 2) and did not differ based on the type of transplanted organ (Fig. 2b). Longitudinal analysis revealed that there was a significant association between SOT status and viral rpM, with SOT recipients demonstrating impaired viral clearance compared to non-SOT controls (Fig. 2c, P = 0.0022).

### Immune cell populations and SARS-CoV-2 antibody levels

**Figure 3:**
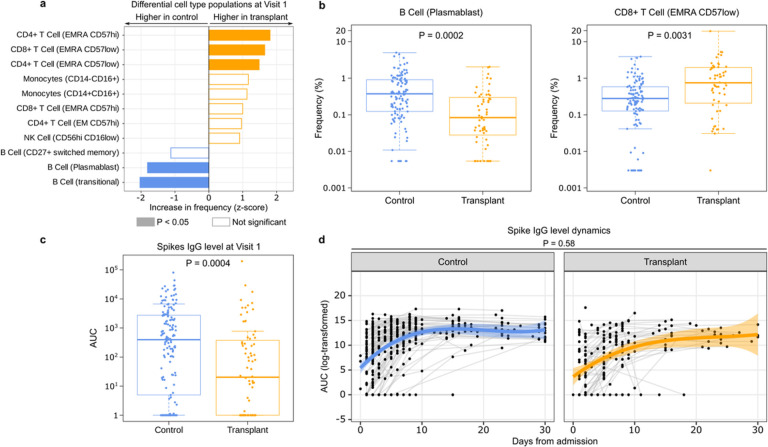
Compared to controls, SOT recipients have lower B cell plasmablasts and higher EMRA T cells as well as lower SARS-CoV-2 antibody levels at hospitalization. (a) Differences in immune cell population frequency measured by CyTOF by SOT recipients and controls. (b) Boxplots highlighting two cell types which differed in frequency between SOT recipients and controls. (c) Boxplot of spike IgG levels measured by area under the curve (AUC). (d) Longitudinal dynamics of spike IgG levels (log-transformed AUC) in SOT recipients and controls over the course of hospitalization. The ribbons indicate the 95% confidence interval of the generalized additive mixed models fit. P-values were calculated with (a–c) a linear model or (d) a linear mixed effects model, with Benjamini-Hochberg correction.

To measure immune cell populations in blood, we used mass cytometry (CyTOF) with a panel of 43 antibodies designed to identify cell lineages and markers of functional status. In PBMC samples from Visit 1, we found 5 cell types associated with SOT status (FDR < 0.05) (Fig. 3a). Plasmablasts and transitional B cells were significantly less abundant at Visit 1 in SOT recipients compared to controls (Figs. 3a,b). Conversely, SOT recipients demonstrated increased proportions of CD4+ T (EMRA CD57hi) and CD4+ T (EMRA CD57low) cells, and CD8+ T (EMRA CD57low) cells, associated with senescence^19^ (Figs. 3a,b). After adjusting for SARS-CoV-2 viral rpM, only plasmablasts and CD4+ T (EMRA CD57low) cells remained statistically significant in terms of proportional differences, suggesting that these two cell types were associated with SOT status in a viral rpM-independent manner (Supp. Fig. 3).

We also compared anti-SARS-CoV-2 spike IgG levels between groups. SOT recipients had lower antibody levels at Visit 1 (P = 0.0004, Fig. 3c), although the rates of increase did not differ based on SOT status (Fig. 3d).

### Cytokine and chemokine expression upon hospitalization and over time

**Figure 4:**
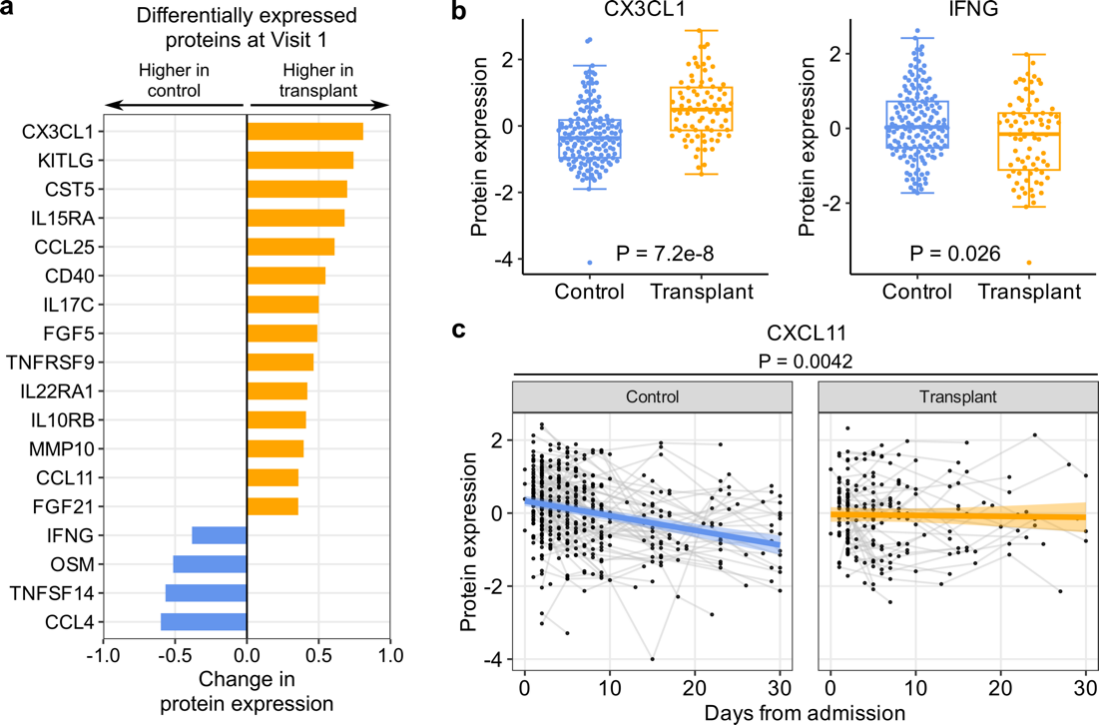
SOT recipients have higher levels of specific serum chemokines and lower levels of IFN-gamma. (a) Bar plots showing proteins that are differentially expressed between control and transplant patients at Visit 1 (adjusted P < 0.05). (b) Box plots showing the levels of CX3CL1 and IFNG at Visit 1. In (a, b), P-values were calculated using a linear model and Benjamini-Hochberg correction. (c) Scatter plot showing the dynamics of CXCL11 level after hospital admission (without adjusting for SARS-CoV-2 viral rpM). The ribbons indicate the 95% confidence interval of the linear mixed effects model fits. P-value was calculated using a linear mixed effects model and Benjamini-Hochberg correction.

Analysis of proximity extension assay (Olink) proteomics data from serum samples identified 16 proteins differentially expressed based on SOT status at hospital admission (Fig. 4a). The expression levels of 12 (75%) of these proteins were higher in SOT recipients versus non-SOT controls, including CX3CL1, IL15RA and KITLG, (Fig. 4b). SOT recipients had lower levels of IFN-gamma (IFNG), OSM, TNSF14, and CCL4. To assess whether differences in SARS-CoV-2 viral rpM may contribute to the observed differential protein expression, we repeated the analyses with adjustment for viral rpM. We found that the results changed minimally, suggesting that SARS-CoV-2 viral rpM did not significantly affect the differential protein expression between SOT recipients and controls (Supp. Fig. 4a).

Analysis of longitudinal serum cytokine expression dynamics revealed that the IFN-inducible chemokine CXCL11 decreased significantly over time in controls, but not in SOT recipients (Fig. 4c). After adjusting for viral rpM differences, CXCL11 dynamics remained significantly different between the two groups, along with a more rapid rise over time in CCL3 and CCL4 expression in the SOT recipients compared to the controls (Supp. Fig. 4b).

### PBMC gene expression differences upon hospitalization, and over time

**Figure 5:**
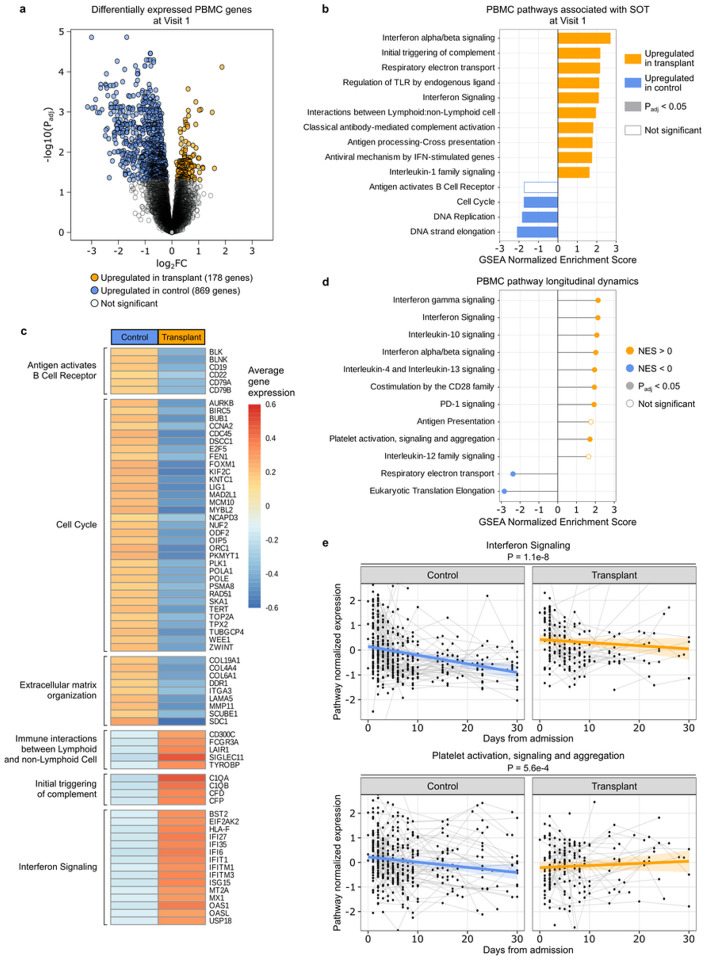
PBMC transcriptomics demonstrates that SOT recipients exhibit increased innate immune gene expression upon hospitalization, and over time. (a) Volcano plot highlighting genes differentially expressed (P_adj_ < 0.05) between SOT recipients and controls at the time of hospitalization. (b) GSEA highlighting pathways differentially enriched in SOT recipients versus controls (without adjustment for SARS-CoV-2 viral rpM). (c) Average gene expression plot of leading-edge genes from significant GSEA pathways. (d) Differences in the longitudinal dynamics of signaling pathways. (e) Longitudinal plots highlighting changes in normalized expression of representative immune signaling pathways that significantly differed over time in SOT recipient versus controls. The ribbons indicate the 95% confidence interval of the linear mixed effects model fits. P-values were calculated with (a) a linear model or (b,d–e) a linear mixed effects model with Benjamini-Hochberg correction.

At the time of hospital admission, differential expression analysis revealed 1047 differentially expressed genes (P_adj_ < 0.05) between SOT recipients and controls (Fig. 5a, Supp. Data 1). Gene set enrichment analysis (GSEA) demonstrated that SOT recipients had increased expression of innate immunity pathways related to type I IFN, TLR signaling, complement activation, IL-1 signaling, and other functions. SOT recipients also exhibited lower expression of B-cell receptor signaling and cell cycle-related pathways (Fig. 5b, c). Adjusting for SARS-CoV-2 viral rpM in the differential expression analysis demonstrated that the increased expression of type I IFN and IL-1 signaling pathways were independent of viral rpM (Supp. Fig. 5a)

We next evaluated the dynamics of gene expression over the course of hospitalization in SOT recipients and controls. SOT recipients exhibited increased expression over time of genes related to several immune pathways including types I and II IFN, IL-10, and PD-1 signaling, and CD-28 co-stimulation (Fig 5d, Supp. Data 2). Adjusting for viral rpM did not significantly affect results (Supp. Fig. 5b). Some signaling pathways (e.g., interferon signaling) decreased more strongly over time in non-SOT controls compared to SOT recipients (Fig. 5e). For other pathways (e.g., platelet activation, signaling and aggregation), SOT recipients demonstrated pathway upregulation over time, while in the control group downregulation was observed (Fig. 5e, Supp. Fig. 5c, Supp. Data 3).

### Upper respiratory tract gene expression differences between SOT recipients and controls

**Figure 6:**
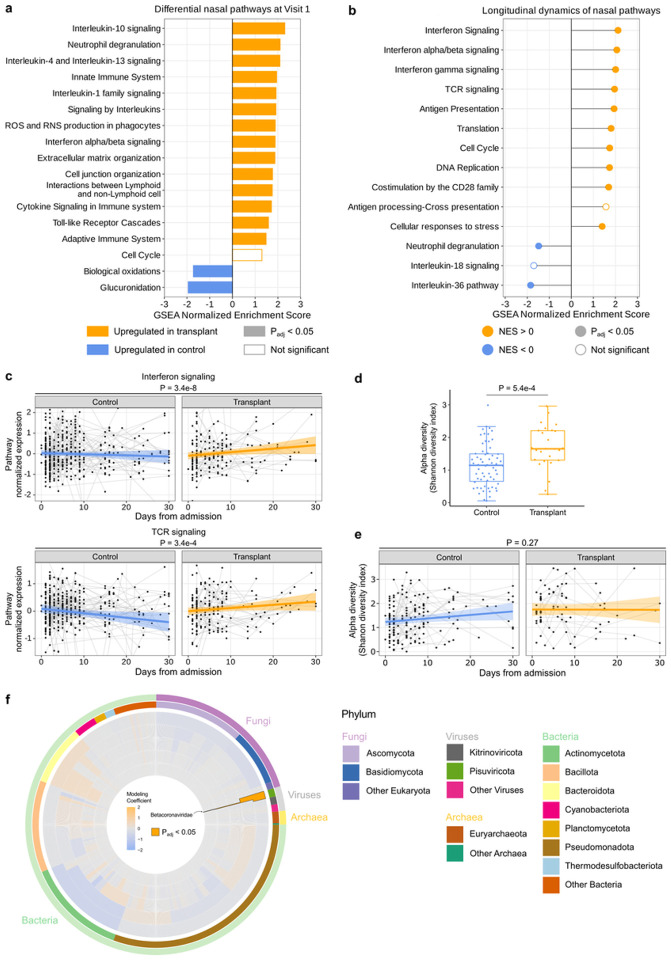
Upper airway host gene expression and the nasal microbiome differ between SOT recipients and controls. (a) GSEA highlighting pathways differentially enriched in SOT recipients versus controls in the upper respiratory tract (without adjustment for SARS-CoV-2 viral rpM). (b) Differences in the longitudinal dynamics of signaling pathways. A positive NES value indicates that the pathway was enriched over time in SOT. A negative NES value indicates that the pathway was enriched over time in controls (c) Longitudinal plots highlighting changes in normalized expression of representative immune signaling pathways that showed significantly different dynamics in SOT recipient versus controls. The ribbons indicate the 95% confidence interval of the linear mixed effects model fits. P-values were calculated with (a) a linear model or (b–c) a linear mixed effects model with Benjamini-Hochberg correction. (d) Box plot demonstrating differences in upper airway bacterial microbiome alpha diversity in SOT recipients versus controls. e) Robust regression with 95% confidence intervals highlighting the longitudinal changes in upper airway alpha diversity following hospitalization. (f) Radial plot highlighting differential abundance from genus (inner most ring) to phylum (outer most ring) and phylogenetic relatedness (inner tree) of taxa differentially enriched in SOT recipients versus controls.

Recognizing that the respiratory tract is the site of active infection in COVID-19, we performed gene expression analyses from nasal swab specimens.Surprisingly, despite the significant difference in the nasal viral rpM (Fig. 2a), no differentially expressed genes were identified between groups at a false discovery rate (FDR) < 0.05 at the time of hospital admission. GSEA nonetheless demonstrated that SOT recipients exhibited increased expression of genes related to IL-10 signaling, neutrophil degranulation, type I IFN signaling, IL-1, and IL-4/IL-13 signaling in the upper respiratory tract at the time of hospital admission (Fig. 6a, Supp. Data 4), mirroring to some extent our observations in the blood. Most inflammatory pathways differentially upregulated in SOT recipients were unaffected by viral rpM adjustment (Supp. Fig. 6a).

Similar to observations in blood, longitudinal nasal transcriptional profiling analyses demonstrated increased expression over time of genes related to IFN signaling (Figs. 6b, c), TCR signaling (Figs. 6b, 6c), PD-1, IL-4, IL-13, and other immune signaling pathways in SOT recipients. In contrast, non-SOT controls demonstrated increased expression over time of genes related to neutrophil degranulation and IL-36 signaling (Fig. 6b, Supp. Data 5). Adjusting for viral load did not significantly change results (Supp. Fig. 6b).

Taken together, these results suggested that SOT recipients, in both the upper respiratory tract and the blood compartments, exhibit augmented innate immune responses at the transcriptional level compared to non-SOT controls, with some compartment-specificity to the relevant immune signaling pathways.

### Differing relationships between interferon signaling and viral abundance in SOT recipients versus controls

In both the blood and the upper respiratory tract, SOT recipients exhibited increased type I IFN gene expression in a viral rpM-independent manner (Supp. Fig. 5a, S6a). We further explored this by comparing the relationship between IFN-stimulated gene (ISG) expression and viral rpM in SOT recipients versus non-SOT controls (Supp. Fig. 7). In blood, ISG expression strongly correlated with viral rpM in non-SOT controls, but this relationship was weaker in the SOT recipients (Supp. Fig. 7a,c). In contrast, in the upper respiratory tract, ISGs correlated with viral rpM in both groups (Supp. Fig. 7b, d). Together, these results suggested a decoupling of peripheral innate immune responses and SARS-CoV-2 challenge in SOT recipients compared to controls.

### Airway microbiome differences between SOT recipients and controls

Next, we used nasal metatranscriptomics to assess whether the composition of the respiratory microbiome differed between SOT recipients and controls upon hospital admission. We found that SOT recipients had greater upper airway microbiome alpha diversity, as measured by Shannon Diversity Index (SDI), compared to controls at Visit 1 (Fig. 6d,e). No differences in bacterial community composition (beta diversity) between groups, as measured by the Bray Curtis Dissimilarity Index, were found at Visit 1 (P=0.179, Supp. Fig. 8).

### Immune correlates of COVID-19 severity differ between SOT recipients and controls

We characterized differences in host correlates of COVID-19 severity^14–16^ between SOT recipients and non-SOT controls by comparing these groups with respect to cell type frequencies, gene expression, and protein expression differences between patients with severe COVID-19 (TG 4–5) versus those with mild/moderate COVID-19 (TG 1–3).

**Figure 7:**
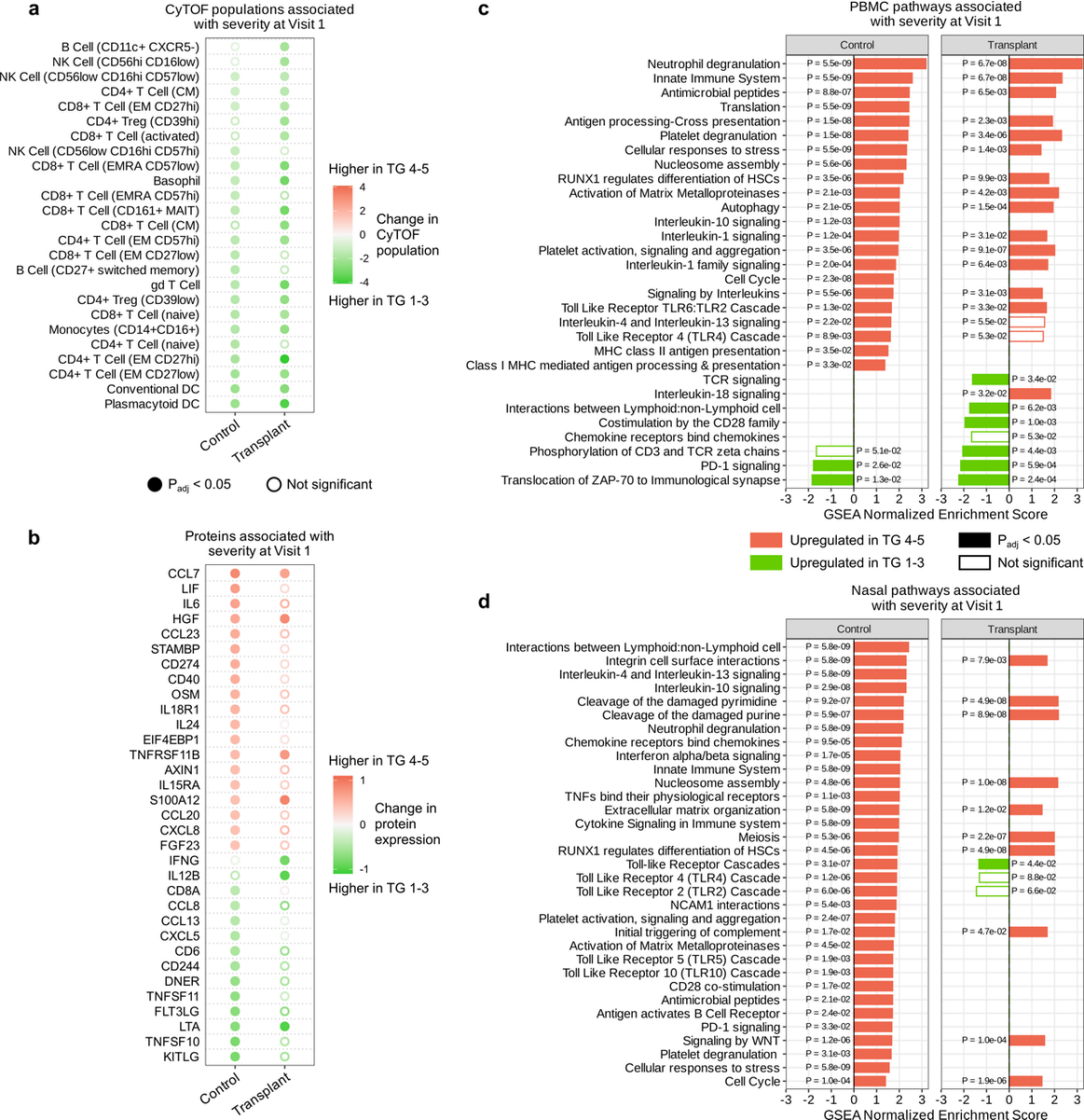
Host immune correlates of COVID-19 severity differ between SOT recipients and controls. (a) Dot plot of immune cell populations that are up- or downregulated in severe patients (TG 4–5) compared to mild/moderate patients (TG 1–3) within each of the control and transplant groups. (b) Dot plot of proteins that are up- or downregulated in severe compared to mild/moderate patients within each of the control and transplant groups. (c) Plots highlighting GSEA-identified signaling pathways from PBMC transcriptomics that were differentially upregulated in severe versus mild/moderate COVID-19 in SOT recipients (right) or controls (left). (d) Plots highlighting GSEA-identified signaling pathways from nasal transcriptomics that were differentially upregulated in severe versus mild/moderate COVID-19 in SOT recipients (right) or controls (left).

In both SOT recipients and controls, severe disease was characterized by reductions in several immune cell populations, including conventional dendritic cells (DCs), and intermediate (CD14+CD16+) monocytes (Fig. 7a). Only SOT recipients, however, had significantly lower CD11C+ CXCR5- B cells in severe disease. Severe non-SOT control patients, in contrast, had lower proportions of naïve CD4+ T cells and CD8+ NKT cells (Fig. 7a). Severe COVID-19 in controls, but not SOT recipients, was associated with a marked increase in several canonical proinflammatory serum cytokines and chemokines (e.g., IL6, CCL7, and CXCL9) (Fig. 7b). Conversely, serum levels of IFNG and IL12B were significantly lower in severe SOT recipients, but not in controls.

A similar analysis of PBMC transcriptomics data revealed that both SOT recipients and controls exhibited greater expression of several immune signaling pathways in severe disease, including neutrophil degranulation, innate immune system signaling, and cellular responses to stress (Fig. 7c). The expression of PBMC genes related to PD-1 signaling decreased in both groups as well. SOT recipients, however, demonstrated lower expression of genes related to TCR signaling, CD28 signaling, and TNF receptor-ligand interactions (Fig. 7c).

Even more notable differences between groups were observed in severity analyses of the upper airway data. Of note, severe disease in controls was characterized by increased expression of genes related to Toll-like receptor (TLR) signaling, whereas SOT recipients demonstrated the opposite (Fig. 7d). SOT recipients did not demonstrate the increases in expression of genes related to neutrophil degranulation, IL-10, IL-4/IL-13, and innate immune signaling observed in controls.

## Discussion

**Figure 8:**
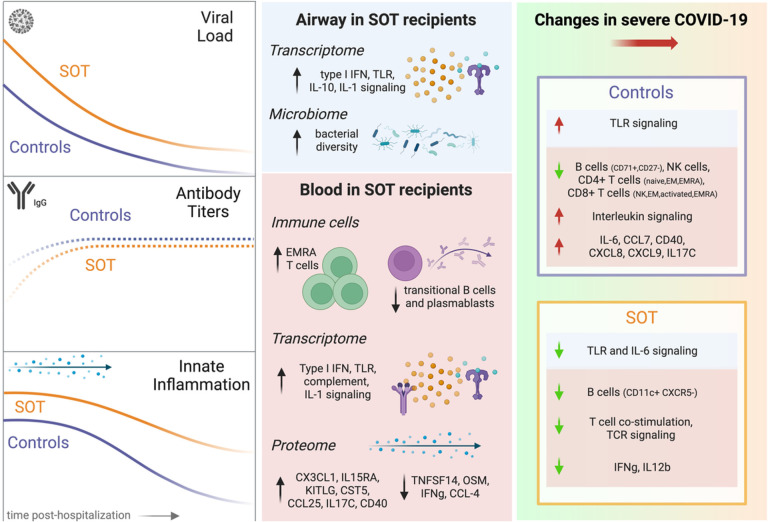
Summary schematic highlighting the unique program of inflammatory dysregulation in SOT recipients hospitalized for COVID-19 based on host/microbe multi-omic profiling.

Pharmacologic immunosuppression is necessary to prevent rejection following SOT, but comes at the expense of increased vulnerability to infection. While it is well known that SOT recipients can exhibit clinically atypical responses to respiratory infections including COVID-19^20^, the molecular features of these differences have remained unclear. Here, we performed comparative host/microbe systems immunoprofiling of SOT recipients and matched non-SOT controls to address this key knowledge gap. Unexpectedly, we found that COVID-19 in SOT recipients is not characterized by globally suppressed systemic immune signaling, but instead by augmented innate immune responses and more subtle differences across states of COVID severity (Fig. 8).

In the peripheral blood of SOT recipients, augmented innate immune signaling was characterized by higher expression of genes related to type I IFN, IL-1, and complement system pathways. Throughout the course of hospitalization, SOT recipients demonstrated consistent increases in these inflammatory signaling pathways, as well as in PD-1 and CD28 signaling. At the protein level, SOT recipients had higher levels of a few proinflammatory cytokines, such as CX3CL1, a potent chemoattractant of T cells and monocytes, and KITLG, which plays a role in hematopoiesis. In addition, SOT recipients demonstrated an increase in CXCL11 levels over time. Together, these results highlight an unexpected state of activated innate immune signaling in SOT recipients at the time of hospitalization, complemented by stable to increased expression of *PD1* and other genes related to T cell signaling and exhaustion over the course of hospitalization.

We found that this state of innate immune activation was driven in part by higher SARS-CoV-2 viral load in SOT recipients, as adjustment for SARS-CoV-2 rpM impacted the magnitude of expression differences for some proinflammatory signaling pathways and cytokines. Most notable was type I IFN signaling, reflecting the proportional induction of many ISGs in response to viral RNA levels^21,22^. The lower proportions of naïve B and T cell populations in SOT recipients that we observed by mass cytometry presumably contributed to impaired control of viral replication, and the downstream activation of innate immune pathways.

In longitudinal analyses, however, even after adjusting for viral rpM differences, SOT recipients demonstrated consistently greater induction of innate immune signaling pathways in the peripheral circulation, including type I IFN signaling, compared to non-SOT controls. Furthermore, while ISG expression in the blood strongly correlated with viral rpM in non-SOT controls, this relationship was not consistently observed in SOT recipients, suggesting a partial decoupling of IFN signaling and viral pathogen burden.

In the upper airway, transcriptional differences between SOT recipients and controls were subtle, although GSEA did reveal important distinctions between groups at the pathway level. Most notably, as in the blood, SOT recipients demonstrated evidence of upregulated innate immune responses in the airways characterized by increased expression of genes related to type I IFN signaling, IL-1 signaling, and complement activation. In contrast to blood, expression of ISGs in the upper airway was strongly correlated with SARS-CoV-2 viral rpM in both non-SOT controls and SOT recipients.

In line with many prior studies^14–16^, higher expression of proinflammatory cytokines such as IL-6 correlated with disease severity in non-SOT control patients. In contrast, we found that the expression of most inflammatory cytokines in SOT recipients minimally differed between mild/moderate and severe disease. In addition, while controls exhibited marked severity-associated increases in the expression of canonical proinflammatory genes, this was not observed in SOT recipients. Instead, severe disease in SOT recipients was associated with lower T cell signaling gene expression in the blood, as well as less robust induction of TLR and IL-6 signaling pathways in the upper airway (Fig 7). These observations suggest a profound difference in the immune milieus in SOT versus non-SOT patients, depending on severity. The lack of association between increased proinflammatory serum cytokines in SOT patients and severe COVID-19 may have important implications, and suggests that the clinical utility of immune modulatory therapies, such as IL-6 inhibitors (e.g., tocilizumab), or JAK inhibitors (e.g., barcitinib) may not be the same in SOT recipients as in the general population.

Despite their increased susceptibility to SARS-CoV-2 infection, and comparatively poor outcomes with other respiratory infections^23^, SOT recipients paradoxically have comparable COVID-19 mortality versus the general population, in propensity matched studies^5,8–10^. Our observation that severe disease in SOT recipients is not characterized by a marked increase in mortality-associated inflammatory cytokines such as IL-6, offers a potential explanation. Our findings could also simply reflect higher levels of innate immune signaling in SOT recipients versus controls across many states of disease severity, possibly representing a compensatory effect of immunosuppressant medications that predominantly target the adaptive immune system.

These observations lead to a model of immune perturbation in COVID-19 with very different profiles in SOT recipients compared to non-SOT controls. In SOT patients on chronic immune suppression, increased senescent T cells and decreased plasmablast and B cells are unable to effectively clear virus, leading to increased and persistent viral replication. Perhaps in a compensatory effort, innate immune responses, such as type I interferon, are upregulated, and fail to attenuate appropriately over time. This dysregulated state of impaired B and T cell immunity, delayed viral clearance, and augmented innate immune signaling has parallels with aging-associated inflammatory changes^24^ and may have important implications for management of immunosuppression in SOT patients with acute infection.

Our study has several strengths. These include a large, comparative immunoprofiling study of SOT recipients during their first encounter with a novel viral pathogen without the complication of variable vaccination histories. In addition, our multicenter design, and assessment of both host and microbe using a diverse range of assays are strengths. Our study also has limitations including an insufficient sample size to assess differences based on type of transplanted organ, limited clinical data regarding immunosuppressant dosing, a small sample size of intubated patients with severe COVID-19, and a lack of data specific to allograft function. Further work is needed to determine whether our findings in SOT recipients with COVID-19 also apply to other types of viral, bacterial, and fungal respiratory infections.

Taken together, we find that COVID-19 in SOT recipients is characterized by a biologically distinct immune state with augmented innate signaling but lower proportions of certain adaptive immune cell populations. The distinct immune state of SOT recipients lacks the dynamic induction of genes and cytokines associated with severe COVID-19 in the general population, suggesting a role for unique prognostic biomarkers and therapeutic approaches in this vulnerable population.

## Methods

### Patient enrollment and sample collection

This study leveraged data from the IMPACC cohort^17,18^, which enrolled 1286 participants from 20 hospitals across 15 medical centers in the United States between May 5th, 2020 and March 19th, 2021. Eligible participants were participants hospitalized with SARS-CoV-2 infection confirmed by RT-PCR and symptoms or signs consistent with COVID-19. Solid organ transplant (SOT) patients were identified by review of medication list for immunosuppressive medications. Patients identified as SOT recipients were confirmed by chart review to verify transplant status and organ type. We conducted a case-control study of patients within the IMPACC cohort, matching all 86 solid organ transplant (SOT) recipients 2:1 by age, sex and study site with 172 immunocompetent controls. The detailed study design and schedule for clinical data and biologic sample collection, and shared core platform assessments were previously described^16,18^. Detailed clinical assessments and sampling of blood and upper respiratory tract were performed within ~72 hours of hospitalization (Visit 1), and on approximately Days 4, 7, 14, 21, 28 after hospital admission. As previously described^18^, biological sample collection and processing followed a standard protocol utilized by every participating academic institution.

### Ethics

NIAID staff conferred with the Department of Health and Human Services Office for Human Research Protections (OHRP) regarding potential applicability of the public health surveillance exception [45CFR46.102(l)(2)] to the IMPACC study protocol. OHRP concurred that the study satisfied criteria for the public health surveillance exception, and the IMPACC study team sent the study protocol, and participant information sheet for review, and assessment to institutional review boards (IRBs) at participating institutions. Twelve institutions elected to conduct the study as public health surveillance, while three sites with prior IRB-approved biobanking protocols elected to integrate and conduct IMPACC under their institutional protocols (University of Texas at Austin, IRB 2020-04-0117; University of California San Francisco, IRB 20–30497; Case Western reserve university, IRB STUDY20200573) with informed consent requirements. Participants enrolled under the public health surveillance exclusion were provided information sheets describing the study, samples to be collected, and plans for data de-identification, and use. Those that requested not to participate after reviewing the information sheet were not enrolled. Participants did not receive compensation for study participation while hospitalized, and subsequently were offered compensation during outpatient follow-up.

### Common statistical analyses framework

Deidentified quality assured raw data was obtained from the IMPACC study and made publicly available^17,18^. All data analyses employed R v4.0.2. For each data type, we investigated the behavior of features both at Visit 1 (within 72 hours of hospital admission for most of the participants) and longitudinally for scheduled visits (Visits 1–6, up to 30 days post-hospital admission, both inpatient and outpatient samples, and excluding escalation samples). For Visit 1 analyses, we used linear modeling with age as a continuous variable and controlled for sex and baseline respiratory severity. Longitudinal measures of the WHO 7-point severity ordinal scale over time were clustered into five trajectory groups (TG) using group-based trajectory modeling, a likelihood-based approach commonly used to group time series of clinical data, as described previously^16^. For the severity analysis, we defined mild participants as those with trajectory group (TG) 1–3, and severe participants as those with TG 4–5.

For longitudinal analysis of SARS-CoV-2 nasal viral rpM and serum anti-Spike IgG, we used generalized additive models with mixed effects from the package gamm4 (v0.2.6) to evaluate the effects of age while controlling for sex and TG. Generalized additive modeling was preferred for these features due to their non-linear trajectories as previously reported. For all other data types, we used linear mixed effects models from the package lme4 (v1.1.25). P values in all analyses were adjusted with Benjamini-Hochberg correction.

### Analysis of nasal metatranscriptomics data

Taxonomic alignments from nasal metatranscriptomics data were obtained from raw fastq files using the CZ-ID pipeline^25^, which first removes human sequences via subtractive alignment against human genome build 38, followed by quality and complexity filtering. Subsequently, reference-based taxonomic alignment at both the nucleotide and amino acid levels against sequences in the National Center for Biotechnology Information (NCBI) nucleotide (NT) and non-redundant (NR) databases, respectively, is carried out, followed by assembly of the reads matching each taxon. Taxa were aggregated to the genus and higher phylogenetic levels from NCBI for analyses^26^. For all analyses using SARS-CoV-2 viral rpM, log transformation of total reads per million (rpM) aligned to the Beta-coronavirus genus was used. Alpha diversity (Shannon Diversity Index) was calculated using the vegan package v.2.6 in R. Differential abundance analyses for Visit 1 samples were performed using linear mixed effect modeling (using the R package nlme v3.1–162) to evaluate SOT effect on individual taxon levels at the genus, family, class, order, phylum, and superphylum levels (rpMs from lower taxon levels were summed to create higher phylogenetic level rpM), using the following formula:

Taxon_abundance~transplant_statuswithrandomeffectsof(1|enrollment_site)

Principle coordinate analysis (PCoA) of Bray-Curtis dissimilarity index was performed on Visit 1 nasal metatranscriptomics samples, with significance calculated with Adonis using the R package vegan (v2.6).

### Analysis of SARS-CoV-2 viral abundance

SARS-CoV-2 viral abundance was calculated as log10(rpM+1), where rpM is the reads per million of SARS-CoV-2 as measured by nasal metatranscriptomics. The viral rpM in each organ transplant type was compared using a likelihood ratio test on the null and alternative models:

Null:viral_rpm~1

Alternative:viral_rpm~organ_type

Where viral_rpm was the log10-transformed viral rpM, and organ_type was the organ transplant type. Longitudinal analysis of SARS-CoV-2 viral rpM was performed using the gamm4 function from the gamm4 package (v0.2.6), using the following formula:

viral_rpm~s(event_date,bs=“cr”)+s(event_date,bs=“cr”,by=transplant)+transplant

with random effects (1|pid), ie, participant random intercept. In the formula, viral_rpm was the log10-transformed viral rpM as described above, event_date was the number of days post hospitalization, transplant is the transplant status. P-value was calculated using the Chi-squared test on the gam component’s reference degrees of freedom and F-statistics.

### Analysis of SARS-CoV-2 antibody titers

Antibody levels against the recombinant SARS-CoV-2 spike protein receptor-binding domain (RBD) were measured using a research-grade enzyme-linked immunosorbent assay (ELISA) as described^16^. The optical density (OD) was measured and the area under the curve (AUC) was calculated, considering 0.15 OD as the cutoff. For the Visit 1 analysis, we log2-transformed the AUC values and modeled them with linear regression. For the longitudinal analysis, we also log2-transformed the AUC values, and used the linear mixed effects models (to fit the null and alternative models:

Null:z~event_date+transplant+(1|pid)

Alternative:z~event_date+transplant+event_date:transplant+(1|pid)

Where z is the log2-transformed AUC, event_date is the number of days post-admission, transplant is the transplant status, and (1|pid) is the participant random intercept. The P-values were calculated using likelihood ratio test, and adjusted with Benjamini-Hochberg correction. For visualization of longitudinal antibody levels, data was fit to a third-order polynomial.

### Analysis of PBMC and nasal RNA-seq data

RNA was extracted from PBMC and inferior nasal turbinate swabs and gene expression levels were quantified by RNA-seq as previously described^17^. For all RNA-seq analyses, we retained protein-coding genes that had a minimum of 10 counts in at least 20% of the samples. We normalized the gene counts using the voom function (normalize.method = “quantile”) from the limma package v3.46.0, fitted a linear model for the gene expression with lmFit function (default settings), calculated the empirical Bayes statistics with eBayes function (default settings), and calculated the P values for differential expression controlling for FDR. We controlled for log-transformed viral rpM in certain analyses when indicated.

For longitudinal analyses, we accounted for repeat measures from the same individual using duplicateCorrelation from the limma package, and modelled the interaction between days post-admission and transplant status as follows:

z~event_date+transplant+event_date:transplant

Where z is the log-transformed normalized expression count, event_date is the number of days post-admission, and transplant is the transplant status.

Fold-change values for Visit 1 analyses, representing the fold-change of transplant patients over control patients, and longitudinal analyses, representing the interaction term of days post-admission and transplant status, were used as the input for Gene Set Enrichment Analysis (GSEA). We used the gsePathway function from the ReactomePA v1.42.0 package to search for enriched pathways in the Reactome database, with minimum and maximum geneSet sizes of 3 and 1000, respectively.

For analysis of the relationship between interferon signaling and viral rpM at Visit 1, we first subset the total PBMC and nasal RNA-Seq data to genes within the Reactome *Interferon Signaling* pathway (R-HSA-913531, n=308). We then split the data by transplant status and modelled the relationship between interferon signaling gene expression and log2-transformed viral rpM for controls and transplant recipients separately, using the approach described above.

### Analysis of CyTOF data

PBMCs were phenotyped on the Fluidigm Helios mass cytometer using a panel of 46 surface and intracellular markers, and the cell types were annotated using an automated annotation pipeline as previously described^16^. Prior to analysis, we removed cells identified as red blood cells, multiplets, debris, and those that were not identifiable with high confidence. These counts were converted to proportions per sample, by dividing each cell type count by the total cell count. The minimum proportion per cell type across all samples was added to each sample prior to log2-transformation, to avoid taking the logarithm of zeros.

For the Visit 1 analysis, the log2-transformed cell type proportions were modeled with linear regression. For the longitudinal analysis, the log2-transformed cell type proportions were modelled with linear mixed effects models (to fit the null and alternative models:

Null:z~event_date+transplant+(1|pid)

Alternative:z~event_date+transplant+event_date:transplant+(1|pid)

Where z is the log2-transformed cell type proportion, event_date is the number of days post-admission, transplant is the transplant status, and (1|pid) is the participant random intercept. The P-values were calculated using likelihood ratio test, and adjusted with Benjamini-Hochberg correction.

### Analysis of serum inflammatory protein (Olink) data

All samples were processed with the Olink multiplex assay inflammatory panels (Olink Proteomics), according to the manufacturer’s instructions and as previously described^16^. This inflammatory panel included 92 proteins associated with human inflammatory conditions. Target protein quantification was performed by real-time microfluidic qPCR via the Normalized Protein Expression (NPX) manager software. Data were normalized using internal controls in every sample, inter-plate control and negative controls, and correction factor and expressed as log2 scale proportional to the protein concentration. For additional quality control, we set any NPX measurements below the assay’s limit of detection (LOD) to zero. Next, we excluded proteins that were detected in fewer than 20% of samples, resulting in 84 proteins for analysis.

For the Visit 1 analysis, we standardized the NPX values and modeled them with linear regression, with and without adjusting for SARS-CoV-2 viral rpM. The viral rpM was also calculated as log10(rpM+1). We then fit the following linear model:

z~transplant+transplant:TG

Where z is the standardized protein level, transplant is the SOT status, and transplant:TG is the interaction term between SOT status and disease severity. This formulation allows us to find two separate coefficients (i.e., two separate log fold-change values) for the effects of severity, one for the SOT group and one for the control group.

For the longitudinal analysis, we also standardized the NPX values, and used the linear mixed effects models to fit the null and alternative models:

Null:z~event_date+transplant+(1|pid)

Alternative:z~event_date+transplant+event_date:transplant+(1|pid)

Where z is the standardized protein level, event_date is the number of days post-hospital admission, transplant is the transplant status, and (1|pid) is the participant random intercept. The P-values were calculated using a likelihood ratio test, and adjusted with Benjamini-Hochberg correction.

## Figures and Tables

**Table 1.** **Clinical and Demographic Features of Cohort**.

	SOT Cases	Non-SOT Controls	P-value
**Median age (IQR)**	57.5 (51.3–64.0)	58.0 (50.8–63.3)	0.644
**Site (%)**			0.189
Boston/BWH	7 (8.1%)	18 (10.2%)	
Case Western	2 (2.3%)	7 (4.0%)	
Emory	5 (5.8%)	12 (6.8%)	
Florida	3 (3.5%)	4 (2.3%)	
ISMMS (Mt Sinai)	1 (1.2%)	2 (1.1%)	
OHSU (Oregon)	2 (2.3%)	3 (1.7%)	
OUHSC (Oklahoma)	2 (2.3%)	0 (0.0%)	
Stanford	2 (2.3%)	12 (6.8%)	
UCLA	40 (46.5%)	54 (30.7%)	
UCSF	15 (17.4%)	47 (26.7%)	
Yale	7 (8.1%)	17 (9.7%)	
**Female sex (%)**	25 (29.1%)	52 (29.5%)	1.000
**Early enrollment (%)**	39 (45.3%)	78 (44.3%)	0.172
**Ethnicity (%)**			0.965
Hispanic or Latino	49 (57.0%)	96 (54.5%)	
Not Hispanic or Latino	36 (41.9%)	79 (44.9%)	
Not Specified	1 (1.2%)	1 (0.6%)	
**Race (%)**			0.393
American Indian/Alaska Native	1 (1.2%)	1 (0.6%)	
Asian	2 (2.4%)	3 (1.7%)	
Black/African American	15 (17.4%)	30 (17.0%)	
Multiple	1 (1.2%)	0 (0.0%)	
Other/Declined	34 (39.5%)	81 (46.0%)	
Unknown/Unavailable	3 (3.5%)	2 (1.1%)	
White	30 (34.9%)	59 (33.5%)	
**Trajectory group**			0.808
1	16 (18.6%)	35 (20.3%)	
2	20 (23.3%)	38 (22.1%)	
3	26 (30.2%)	40 (23.3%)	
4	19 (22.1%)	52 (30.2%)	
5	5 (5.8%)	7 (4.1%)	
**ICU admission (%)**	32 (37.2%)	64 (37.2%)	1.00
**Ever intubated (%)**	17 (19.3%)	42 (23.4%)	0.497
**Mortality (%)**			
D28	5 (5.8%)	7 (4.1%)	0.754
Ever	12 (14.0%)	20 (11.6%)	0.739
**Diabetes (%)**	38 (44.2%)	55 (32.0%)	0.074
**Steroids (%)**	76 (88.4%)	104 (60.5%)	8.3e-6
**Remdesivir (%)**	57 (66.3%)	124 (72.1%)	0.414

## Data Availability

Data files are available at ImmPort under accession number SDY1760 and dbGAP accession number phs002686.v1.p1. All analysis code has been deposited at https://bitbucket.org/kleinstein/impacc-public-code/src/master/SOT_manuscript/.

## References

[1] World Health Organization. WHO Coronavirus (COVID-19) Dashboard. https://covid19.who.int.

[2] Barnes E. SARS-CoV-2-specific immune responses and clinical outcomes after COVID-19 vaccination in patients with immune-suppressive disease. Nat Med 29, 1760–1774 (2023). 37414897 10.1038/s41591-023-02414-4 PMC10353927

[3] Müller T. R. Additive effects of booster mRNA vaccination and SARS-CoV-2 Omicron infection on T cell immunity across immunocompromised states. Sci Transl Med 15, eadg9452 (2023). 37437015 10.1126/scitranslmed.adg9452 PMC7615622

[4] Trapani S. Incidence and outcome of SARS-CoV-2 infection on solid organ transplantation recipients: A nationwide population-based study. Am J Transplant 21, 2509–2521 (2021). 33278850 10.1111/ajt.16428 PMC9906464

[5] Kates O. S. Coronavirus Disease 2019 in Solid Organ Transplant: A Multicenter Cohort Study. Clinical Infectious Diseases 73, e4090–e4099 (2021). 32766815 10.1093/cid/ciaa1097 PMC7454362

[6] Schaenman J. Impact of solid organ transplant status on outcomes of hospitalized patients with COVID-19 infection. Transpl Infect Dis 24, e13853 (2022). 35579437 10.1111/tid.13853 PMC9347588

[7] Azzi Y., Bartash R., Scalea J., Loarte-Campos P. & Akalin E. COVID-19 and Solid Organ Transplantation: A Review Article. Transplantation 105, 37–55 (2021). 33148977 10.1097/TP.0000000000003523

[8] Bartelt L. & van Duin D. An overview of COVID-19 in solid organ transplantation. Clin Microbiol Infect 28, 779–784 (2022). 35189336 10.1016/j.cmi.2022.02.005 PMC8855607

[9] Hadi Y. B., Naqvi S. F. Z., Kupec J. T., Sofka S. & Sarwari A. Outcomes of COVID-19 in Solid Organ Transplant Recipients: A Propensity-matched Analysis of a Large Research Network. Transplantation 105, 1365–1371 (2021). 33988341 10.1097/TP.0000000000003670 PMC8414593

[10] Linares L. A propensity score-matched analysis of mortality in solid organ transplant patients with COVID-19 compared to non-solid organ transplant patients. PLoS One 16, e0247251 (2021). 33657157 10.1371/journal.pone.0247251 PMC7928439

[11] Zhu L. Changes of NK cell subsets with time post-transplant in peripheral blood of renal transplant recipients. Transpl Immunol 49, 59–71 (2018). 29702201 10.1016/j.trim.2018.04.005

[12] Wang L. Changes in T and B cell subsets in end stage renal disease patients before and after kidney transplantation. Immun Ageing 18, 43 (2021). 34749733 10.1186/s12979-021-00254-9 PMC8574047

[13] Schaenman J. M. Increased T cell immunosenescence and accelerated maturation phenotypes in older kidney transplant recipients. Hum Immunol 79, 659–667 (2018). 29913200 10.1016/j.humimm.2018.06.006 PMC6429965

[14] Blanco-Melo D. Imbalanced Host Response to SARS-CoV-2 Drives Development of COVID-19. Cell 181, 1036–1045.e9 (2020). 32416070 10.1016/j.cell.2020.04.026 PMC7227586

[15] Koutsakos M. Integrated immune dynamics define correlates of COVID-19 severity and antibody responses. Cell Rep Med 2, 100208 (2021). 33564749 10.1016/j.xcrm.2021.100208 PMC7862905

[16] Arce Joann Multi-omic longitudinal study reveals immune correlates of clinical course among hospitalized COVID-19 patients. Cell Reports Medicine (2023). 10.1016/j.xcrm.2023.101079 PMC10203880 37327781

[17] Ozonoff A. Phenotypes of disease severity in a cohort of hospitalized COVID-19 patients: Results from the IMPACC study. EBioMedicine 83, 104208 (2022). 35952496 10.1016/j.ebiom.2022.104208 PMC9359694

[18] IMPACC Manuscript Writing Team & IMPACC Network Steering Committee. Immunophenotyping assessment in a COVID-19 cohort (IMPACC): A prospective longitudinal study. Sci Immunol 6, eabf3733 (2021). 34376480 10.1126/sciimmunol.abf3733 PMC8713959

[19] Callender L. A. Human CD8 + EMRA T cells display a senescence-associated secretory phenotype regulated by p38 MAPK. Aging Cell 17, e12675 (2018). 29024417 10.1111/acel.12675 PMC5770853

[20] Cheng G.-S. Immunocompromised Host Pneumonia: Definitions and Diagnostic Criteria: An Official American Thoracic Society Workshop Report. Annals ATS 20, 341–353 (2023). 10.1513/AnnalsATS.202212-1019ST PMC9993146 36856712

[21] Mick E. Upper airway gene expression shows a more robust adaptive immune response to SARS-CoV-2 in children. Nat Commun 13, 3937 (2022). 35803954 10.1038/s41467-022-31600-0 PMC9263813

[22] Mick E. Upper airway gene expression reveals suppressed immune responses to SARS-CoV-2 compared with other respiratory viruses. Nature Communications 11, 5854 (2020). 10.1038/s41467-020-19587-y PMC7673985 33203890

[23] Marinelli T. M. & Kumar D. Influenza Virus Infection and Transplantation. Transplantation 105, 968–978 (2021). 33044429 10.1097/TP.0000000000003486

[24] NABEC/UKBEC Consortium The transcriptional landscape of age in human peripheral blood. Nat Commun 6, 8570 (2015). 26490707 10.1038/ncomms9570 PMC4639797

[25] Kalantar K. L. IDseq-An open source cloud-based pipeline and analysis service for metagenomic pathogen detection and monitoring. Gigascience 9, giaa111 (2020). 33057676 10.1093/gigascience/giaa111 PMC7566497

[26] Schoch C. L. NCBI Taxonomy: a comprehensive update on curation, resources and tools. Database (Oxford) 2020, baaa062 (2020). 32761142 10.1093/database/baaa062 PMC7408187

